# Methodological Concerns Regarding the Meta‐Analysis of Cranberry Consumption and Blood Pressure

**DOI:** 10.1002/clc.70339

**Published:** 2026-05-08

**Authors:** Shaher Yar, Muhammad Touseef, Ishvah Shashwat Nag

**Affiliations:** ^1^ The University of Lahore University College of Medicine and Dentistry Lahore Pakistan; ^2^ State Non‐Profit Enterprise Danylo Halytsky Lviv National Medical University Lviv Ukraine

**Keywords:** ambulatory blood pressure monitoring, blood pressure, cranberry, hypertension, meta‐analysis, subgroup analysis

To the Editor,

We read with interest the systematic review and meta‐analysis by Bahreyni and colleagues [1] on cranberry consumption and blood pressure (BP). The authors are to be commended for assembling 12 randomized trials on a topic of growing public interest, and for the detailed subgroup analyses that help frame the clinical debate. The overall finding of non‐significant reductions of roughly 1.3 mmHg in both systolic and diastolic BP is an important contribution to a literature that has been somewhat mixed. We would like to respectfully raise a few points that may help readers interpret the subgroup findings and inform future work in this area.

First, we noticed what appears to be a typographical inconsistency in Table 3 that the authors may wish to clarify. For DBP stratified by duration, the ≤ 8 week row lists a weighted mean difference of −1.59 mmHg (95% CI −2.59 to −0.85) with *p* = 0.21, and the > 8 week row lists −0.99 mmHg (95% CI −2.56 to 0.57) with *p* = 0.002. A confidence interval that excludes zero should not yield a non‐significant p, and vice versa, so we wondered whether the two *p* values may have been transposed. A brief clarification or erratum would help readers identify which duration stratum reached statistical significance.

Second, the overall subgroup pattern may benefit from cautious interpretation. Significant SBP reductions appear in the shorter‐duration stratum while significant DBP reductions appear in the longer, and reductions seen in female‐only trials are not mirrored in the mixed‐sex trials that contain most of the female participants. With 12 trials distributed across five effect modifiers, and in the absence of formal interaction testing or multiplicity adjustment, we would gently suggest that these findings be treated as hypothesis‐generating [2] rather than confirmatory.

Third, the cranberry interventions themselves vary considerably across the included studies, ranging from juices of differing strengths (400–500 mL) to freeze‐dried powder (9 g) and capsules (144–500 mg). Proanthocyanidin (PAC) and anthocyanin content differs widely across these formats, with prior work suggesting a clinically relevant PAC threshold around 72 mg/day [3]. A dose–response meta‐regression by mg PAC/day might offer more mechanistic insight than the “juice versus powder” contrast, and we offer this as a suggestion for future syntheses.

Fourth, only 1 of the 12 included trials, Richter et al. [4] enrolled participants with elevated BP at entry; the remainder recruited normotensive populations or those with other comorbidities. An additional subgroup analysis by baseline BP category could strengthen the interpretation of pooled effects in the populations most likely to benefit clinically.

Finally, the reliance of included trials on office BP, while understandable given the historical literature, may limit sensitivity to small dietary effects. The 2025 ACC/AHA hypertension guideline [5] now recommends ambulatory or 7‐day home BP monitoring for both diagnosis and response assessment, and we hope future trials in this area will adopt these more sensitive endpoints.

Overall, Bahreyni and colleagues have provided a useful framework for thinking about cranberry polyphenols and BP, and their work points clearly to the methodological refinements that would most advance the field. We thank them for their contribution and for the opportunity to comment.

## Author Contributions

All three authors contributed to conception of the letter, critical appraisal of the original meta‐analysis, drafting, and revision, and all approved the final version for submission.

## Conflicts of Interest

The authors declare no conflicts of interest.

## Previous Presentation

This correspondence has not been presented or published elsewhere, in whole or in part.

## In Response To

Bahreyni LZ, Amini MR, Sheikhi L, et al. The effect of cranberry consumption on blood pressure: a systematic review and meta‐analysis of randomized controlled trials. *Clin Cardiol*. 2026;49(4):e70254. doi:10.1002/clc.70254.

## Data Availability

The authors have nothing to report.
